# Influence of Sample Mass and Pouring Temperature on the Effectiveness of Thermal Analysis for Estimating Gray Iron Inoculation Potential

**DOI:** 10.3390/ma18153640

**Published:** 2025-08-02

**Authors:** Raymundo del Campo-Castro, Manuel Castro-Román, Edgar-Ivan Castro-Cedeno, Martín Herrera-Trejo

**Affiliations:** Ingeniería Metalúrgica, Cinvestav Unidad Saltillo, Av. Industria Metalúrgica 1062, Parque Industrial Saltillo-Ramos Arizpe, Ramos Arizpe C.P. 25900, Coahuila, Mexico; raymundo.delcampo@cinvestav.mx (R.d.C.-C.); edgar.castro@cinvestav.mx (E.-I.C.-C.); martin.herrera@cinvestav.edu.mx (M.H.-T.)

**Keywords:** thermal analysis, inoculation potential, melt quality, cast irons

## Abstract

Thermal analysis (TA) has been a valuable tool for controlling the carbon equivalent (CE) of cast irons. Additionally, this technique can provide enhanced control over melt quality, allowing for the avoidance of defects such as undesirable graphite morphology and the formation of carbides. To obtain the most valuable information from the TA, it is necessary to minimize the variations in the filling operation of the TA cups. However, the mass and pouring temperature of TA cups can vary in TA’s typical foundry operations. A design of experiments was performed to determine whether specific parameters of cooling curves used for quality control can distinguish the inoculation effect in the melt when the mass and the pouring temperature of TA cups are varied. The minimum temperature of the eutectic arrest proved to be a robust inoculation potential control parameter when variations in the cup’s mass were within a range of 268–390 g and were filled at any pouring temperature between 1235 and 1369 °C. Lighter cups under 268 g and poured at a low temperature are not suitable for controlling inoculation potential by TA; however, they remain helpful in controlling CE. These later cups are related to cooling times of less than 180 s, which can serve as a criterion for discarding unsuitable samples. A bimodal population of cell surfaces was revealed in the samples, with the population of small cells being proportionally more numerous in samples with lower TE_min_ values.

## 1. Introduction

For decades, industrial thermal analysis (TA) in sand cups has been employed to control metallurgical properties in cast iron foundries [1,2,3]. The first application of TA in cast iron foundries, which has proven remarkably effective, has been in determining CE values and C and Si contents. For such applications, TA-standardized cups are used: plain cups to determine only CE values and cups with tellurium to provide C and Si contents as well. Furthermore, using such an approach, it is implicitly assumed that the undercooling associated with the solidification kinetics of the austenite and the white eutectic remains the same between alloys. Consequently, cooling curve features linked with these constituents can be related to the cast iron’s composition. This approach has demonstrated comparable efficacy to the combustion analysis technique in C and CE determination [4].

Furthermore, thermal analysis has seen increased adoption in foundry applications due to its capability to deliver faster results. However, the relationship of CE, C, and Si with thermal curves could change with Si content, as reviewed recently by Lacaze et al. [5,6], and cup type: square, round, and plain or with tellurium [7]. Then, some calibration is still needed to enhance the precision of CE, C, and Si values obtained through thermal analysis (TA) in specific foundry environments. While CE determination by TA has the advantage of its rapidity, the importance of controlling other elements in the composition of cast iron promotes the search for improvements in sampling techniques for traditional chemical analysis [8].

The use of TA for microstructural control has been developing since the pioneering work in the 1970s, when the relationship between thermal curve features and microstructural aspects was studied [9,10,11], as reviewed by Stefanescu [12]. The need for better control of the quality of cast irons promotes the search for relationships between thermal curves and certain casting features. Schüssler and Bühring-Polaczek found a correlation between specific thermal curve parameters and mechanical properties in ductile irons [13]. This kind of relationship was also sought by Dorula et al. in gray irons [14]. Cree et al. examined the effect of boron and titanium on several casting quality characteristics and how these can be related to the thermal curve characteristics of plain TA cups with two geometries: square and round [15]. Dorula et al. utilized thermal analysis to control porosity formation in gray iron castings [16]. The fading of inoculant in gray irons was estimated by thermal analysis by Jelink et al. [17]. Additionally, thermal analysis was employed by Yu and Whale to assess melt conditions related to shrinkage tendency [18]. The control of carbide formation in gray iron castings by thermal analysis was searched by Neacsu et al. [19].

Several thermal analysis software manufacturers have recently proposed systems for predicting microstructural defects, i.e., assessing the metallurgical quality of liquid metal before pouring, based on the cooling curves of standardized thermal analysis cups. Most industrial thermal analysis devices claim to be capable of assessing the “metallurgical quality” of cast iron alloys; however, the term “metallurgical quality” has a broad spectrum of meanings.

In gray irons, good metallurgical quality is generally defined by a microstructure with a distribution of graphite A uniformly dispersed in flake sizes that depend on the application. Additionally, it is crucial to determine the susceptibility of cast iron to form defects, such as shrinkage, swelling, or carbides. Carbides can be avoided through good inoculation practices. Some process variations, such as the maximal temperature reached during the melting process and its holding time, can affect the effectiveness of the inoculation. For example, a high value of the minimal temperature in the eutectic recalescence, TE_min_, is associated with a graphite distribution A and lower chilling tendency [20]. Also, the possibility of forming carbides increases when the difference between TE_min_ and the white eutectic temperature decreases [21,22]. TE_min_ was determined by Suárez et al. [20] as the more influential thermal analysis parameter compared with the white eutectic temperature to determine the chilling tendency of gray iron.

Ideally, the best condition to extract solidification-related information from cooling curves is to put the thermocouple in the hot spot where there is any heat transfer from the other locations of the TA cup. However, this hot spot could change along with the symmetric axis of the TA cups during the solidification of the cast, as found by simulation by Diószegui et al. [23]. Furthermore, other than the solidification behavior of the alloy, the cooling curve could also be affected by changes in the heat transfer conditions of metal solidifying in TA cups. Then, attention must be paid to avoiding or decreasing heat transfer variations that affect the thermal curve’s shape.

In industrial settings, several factors influence heat transfer and, consequently, the solidification of the sample. These factors include cup filling, pouring temperature, and cup inclination. Sillén [24] (as cited by Stefanescu et al. [25]) noted that the estimation of the CE by the austenite solidification temperature can be affected by cooling rate and nucleation in ductile irons. Stefanescu also identified cup mass and pouring temperature as critical parameters, as they impact cooling rates and solidification kinetics [26]. In industrial applications, weighing TA cup samples is impractical; therefore, this mass effect must be inferred from cooling curves in some way. One study that took into account the inherent effect of the cup’s mass on thermal curves was previously conducted by Suárez et al., who experimented with four different cup sizes [27]. However, they analyzed the data considering only the size variation of the cup, without taking into account the inherent mass changes, as is done in this research, which focuses on a single cup size with different filling levels, as can happen in industrial practice. Our study is then limited to changes in cooling rates that can be induced by varying filling levels in the same type of cup.

Therefore, research was conducted to enhance the understanding of the effects of filling level and pouring temperature on typical cooling curve parameters, which are intended to control the quality of liquid metal. To vary the inoculation potential of the liquid metal, TA cups were filled with inoculated or non-inoculated metal. In doing so, it was possible to test the robustness of some typical thermal analysis parameters to identify the inoculated metal from the non-inoculated metal, even if variations in the tested filling parameters occur.

## 2. Materials and Methods

### 2.1. Design of Experiments

The casting temperature of TA cups was fixed at two levels labeled as high or low temperature. Furthermore, two filling levels of TA cups were considered in this study: a full level, where the cup was filled to its maximum capacity, and a non-full level, which was intended to be filled at approximately 80% of its maximum capacity. To avoid full TA cups, the height of one side of the cup’s top border was reduced by 8 mm. These variations in mass cup and pouring temperature should be reflected in variations in cooling rate. In industrial applications, weighing TA cup samples is impractical. However, the overall heat balance indicates that the cooling rate or an equivalent parameter in thermal curves should be a function of the cup’s mass and T_max_; therefore, the cup’s mass can be estimated from cooling curve data, as shown in the next section.

Additionally, two levels of inoculation were applied to TA cups: half were inoculated with 0.1% Superseed inoculant (Elkem ASA, Oslo, Norway), and the other half were poured without inoculation. Inoculation variation was performed to generate a series of cups filled with the same base metal but exhibiting different solidification kinetics, aiming to identify the parameters within the cooling curves that can distinguish between the two sets of samples.

With the available laboratory melting setup, it is possible to pour 3 TA cups from the same melt. A design of experiments (DoE), as shown in Table 1, was performed to maximize the mix of experimental conditions with the same melt. This DoE allows 6 TA cups of each experimental condition at the two tested temperature ranges. Additionally, a sample for chemical analysis was obtained from the laboratory melts.

Table 1 presents the DoE applied to each pouring temperature range tested. The labels for the experimental conditions of TA cups have the following meanings: F (full), NF (non-full), I (inoculated), NI (non-inoculated), H (high temperature), and L (low temperature).

The TA cups from these experiments have a mass that varies within a range of 268–390 g, which is referred to here as the normal mass range (NM). Such weight variation corresponds well to what was sought to match the observed variation in a sample of industrial cups [28]. Farias [28] found that this weight variation leads to significant differences in cooling time, which complicates the compositional control of cast irons by neural models. In addition, to evaluate the effect of lighter cups on TA, four additional melts were performed by stopping the pouring before the cups were filled under non-inoculated and low-temperature conditions. Additionally, during some of the experiments, some of the thermocouples in the cups malfunctioned, necessitating the repetition of these melts to obtain at least six samples in the same experimental conditions.

### 2.2. Experimental Heats

All the TA cups were obtained from the same base metal to minimize compositional fluctuations between trials. This base metal, whose composition is reported in Table 2, was obtained by melting steel scrap and Sorel iron ingot, 20 kg of each, in a medium-frequency induction furnace with a capacity of 40 kg. A recarburizer, sulfur, and ferroalloys were also added for composition adjustment. Table 1 shows the chemical composition of the base metal, which was poured into small molds to obtain ingots of about 1 kg each.

**Figure 1:**
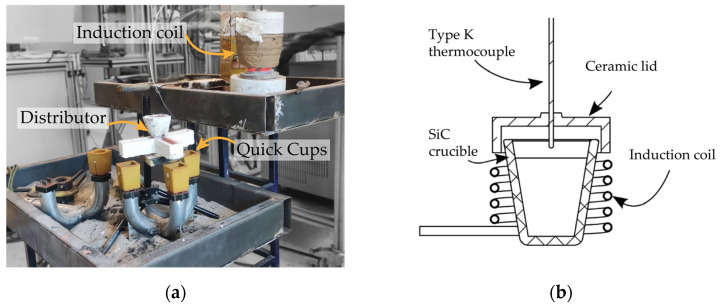
Experimental setup for the heat carried out under the applied DoE: (**a**) general view; (**b**) schematic of the crucible arrangement for metal melting.

These ingots were further melted into a SiC-based crucible in a small induction melter with a maximum capacity of around 2 kg. The experimental setup is shown in Figure 1. The used power source has a 15 kW capacity and operates within the 20–80 kHz frequency range. This experimental setup enabled the efficient melting and pouring of 1.5 kg of iron, which was required to fill three TA cups and pour one sample for chemical analysis. The setup consisted of an A3-sized crucible with a Brimful capacity of 490 cm^3^ and a handcrafted induction coil suitable for this crucible size. A refractory cement cover was also implemented to diminish the melt oxidation and heat transfer losses. The same procedure was meticulously implemented in each melt to maintain homogeneous heating and oxidation conditions throughout the trials. The fusion procedure was as follows: First, the crucible was loaded with one ingot. The second part of the metal was added after the completion of the first ingot’s melting. For heating cycling, power increments of 10% were made every 10 min to reach 40%.

**Figure 2:**
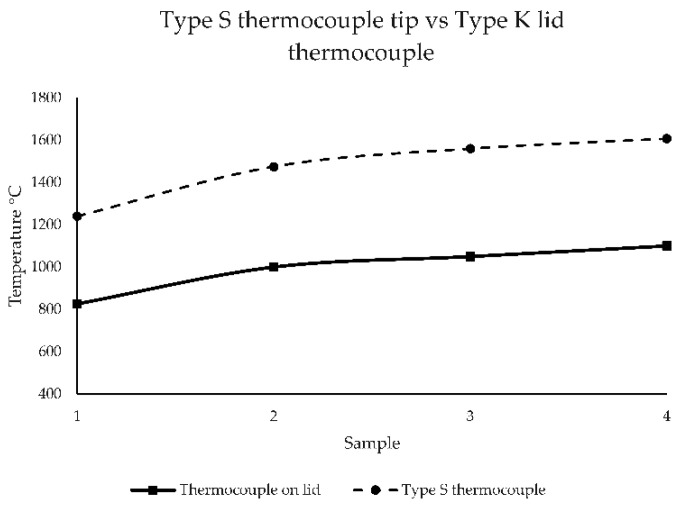
Heating curves comparison: validation measurements using a disposable type S thermocouple and continuous measurements using a type K thermocouple placed on the crucible lid.

The crucible cover was instrumented with a type K thermocouple at the center. This thermocouple was employed to record a reference temperature throughout the entire heating cycle. Preliminary melts were conducted to relate this reference temperature to the melt temperature measured using disposable cartridges with type S thermocouples. The melt temperature was measured at four different times during the melting process. The comparison of these measurements to the reference temperature is shown in Figure 2. The average difference between the two temperature curves was approximately 470 °C. The pouring procedure began when the reference temperatures reached 905 °C or 980 °C, corresponding to estimated melt temperatures of 1375 °C for the lower range and 1450 °C for the higher range. In addition to the effect of pouring temperature on cooling rate, it is also expected that, in the high melt temperature range, the inoculation potential diminishes compared to the low melt temperature range, an effect known in foundry practice [29]. The SiC crucible was extracted from the induction coil after the power source was turned off. This extraction operation and subsequent pouring introduce variations in the time lapses between the power source being turned off and the pouring, allowing a variation in the pouring temperature of the small melt volume. The pouring temperature for each TA cup was related to the maximal temperature recorded by the cup’s thermocouple. For the high-temperature range, two cups had an initial temperature over the measurement range for K thermocouples, i.e., over 1369 °C in our device. In such cases, a value of 1369 °C was considered the pouring temperature. K thermocouples are the most common in TA cups used in the foundry due to their price and temperature measurement range combination. Additionally, their maximum reading temperature can be exceeded for a few seconds without damage.

**Figure 3:**
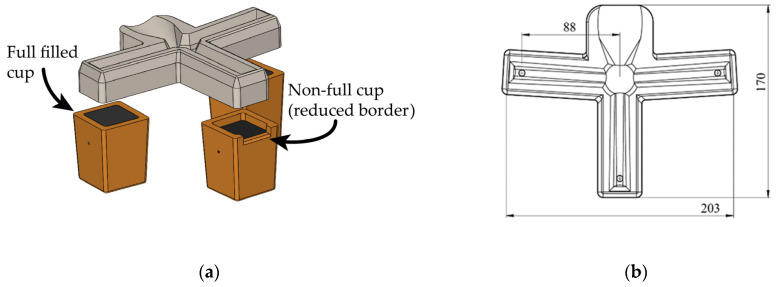
Graphical representation of the ceramic distributor for uniform filling of the cups: (**a**) experimental setup of the distributor and cups; (**b**) top view with dimensions in mm.

Sequentially filling several TA cups using the same spoon is common in thermal analysis experimentation. However, this method increases the differences in the cups’ pour temperature because the pouring temperature decreases gradually as the cups are successively filled. A filling distributor that allows for the simultaneous filling of three cups was implemented to minimize such a temperature difference. Additionally, the inoculant was placed on the pouring channels selected according to the design of experiments (DoE) applied. Inoculated cups do not show any traces of the inoculant, as observed at the bottom of the cups when the inoculant is placed there. This distributor was designed using CAD software and manufactured by casting refractory cement into a 3D-printed mold made of PLA filament (see Figure 3).

**Figure 4:**
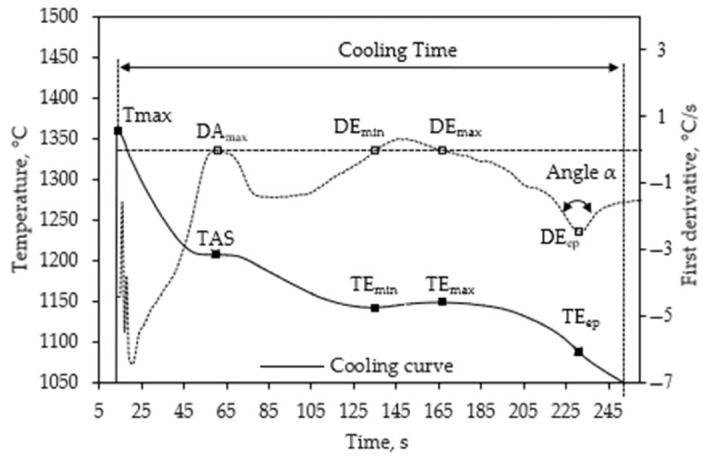
Cooling curve and its first derivative with the definition of the parameters analyzed as a function of mass and pouring temperature of TA cups. The horizontal dashed line represents a zero value of the first derivative, while the vertical dashed line indicates the time corresponding to 1050 °C.

The cooling curves were analyzed to obtain several typical thermal parameters, as shown in Figure 4. The parameters were determined through a comprehensive visual inspection of each cooling curve and its first derivative. In the curve of the first derivative, there are two prominent peaks associated with the slowdown of the cooling rate during solidification. The first corresponds to austenite solidification and the second to the eutectic reaction. Typically, the temperature associated with the maximum of the first peak, DA_max_, is considered the austenite solidification temperature (TAS), which in some cases has been incorrectly referred to as the austenite liquidus temperature (TL). In the eutectic reaction interval, there are three characteristic points in the first derivative. The DE_min_ corresponds to the first zero crossing value in the derivative curve, DE_max_ the zero second crossing value, and DE_ep_ the minimum of the final negative peak observed in the first derivative of the cooling curve during the eutectic reaction. The related temperatures are, respectively, TE_min_, TE_max_, and TE_ep_. Recalescence is the value of the difference between TE_max_ and TE_min_. T_max_ is the maximum temperature measured by the thermocouple right after pouring the TA cup. Cooling time is defined here as the elapsed time from T_max_ to 1050 °C.

TE_ep_ is sometimes considered the temperature at the end of solidification [30,31]. However, the end of solidification cannot be related to DE_ep_ because the part of the period at the end of the solidification peak is due to a transfer lag, which is directly related to thermal diffusivity and conductivity (R. Schumann, cited by Ekpoom and Heine [32]). Ekpoom and Heine observed a negative peak in their simulated curve of differential thermal analysis at this location of the peak. The negative peak was associated with heat absorption, which they considered unrealistic. Also, Castro [33] showed that in thermal curves corresponding to thermocouples located at the center, at the demi-radius, and the mold metal interface of a cylinder, the TE_ep_ is registered at times that increase from the cylinder center to the interface; then, it would also be unrealistic if TE_ep_ is considered as the end of the solidification. However, Ekpoom and Heine mentioned that this peak is of interest to obtain information on the constituents formed during solidification, as they observed that the metastable eutectic peak is smaller than the one for the gray iron eutectic. Keeping in line with Ekpoom and Heine’s suggestion, TE_ep_ is considered here as a parameter that provides an indication of the microstructure formed. Another parameter associated with the deep peak of the eutectic reaction and the microstructure of the castings is the angle α, as shown in Figure 4, or some other values, such as the slopes of lines forming such an angle, which are not considered here. The time elapsed between T_max_ and 1050 °C was labeled in this work as the cooling time (CT).

### 2.3. Eutectic Cell Count

**Figure 5:**
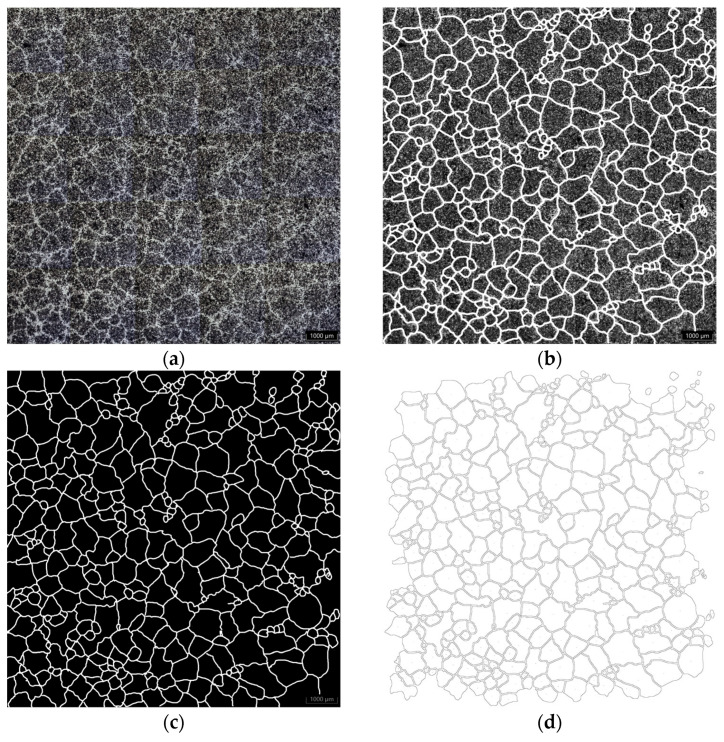
(**a**) Original mosaic of micrographs; (**b**) cell boundary highlighting; (**c**) binary image conversion; (**d**) outlines for counting and surface measurements of cells.

From the group of the TA cups, twelve of them were selected for cell counting. They were chosen to have similar weights and initial temperatures but varied in TE_min_ values. A typical procedure was used for the metallographic preparation of the samples, followed by Stead’s reagent attack to reveal the boundaries between the cells. To count cell numbers, a mosaic of 5 × 5 micrographs taken at 50× magnification in an optical microscope was built. The entire measured surface, 120 mm^2^, was chosen to be close to the thermocouple tip. According to the theory of heterogeneous nucleation, so-called secondary nucleation of cells can occur in some cases at the last stage of solidification. This secondary nucleation must result in two distinct populations of cell sizes. To elucidate if this is possible in our samples, the size of the cells was also measured. To achieve this, the open-source image editing software GIMP 2.9.8 was utilized. The paintbrush tool was applied consistently across all micrographs using identical configurations. Cell boundaries were manually delineated on an independent layer (Figure 5b). The resulting image was subsequently imported into ImageJ 1.54c, where it was first converted to an 8-bit grayscale format and then binarized using the thresholding tool (Figure 5c). Following proper scale calibration, the particle analysis tool was employed to extract cell outlines and to quantify relevant morphological parameters, including area and shape descriptors (Figure 5d).

## 3. Results and Discussion

### 3.1. Chemical Composition

Table 2 shows the composition of the base metal, and eight melts were performed for the DoE. A small loss of C and Si is seen between the base metal and thermal analysis melts. Additionally, it is observed that there is good compositional repeatability among the DoE melts performed.

### 3.2. Thermal Analysis

The values of all parameters obtained from the cooling curves were grouped according to their pouring temperature range and inoculation condition and analyzed as a function of the cup’s mass. In all figures, the data corresponding to the linear regression are also reported. Correlation data with cup mass are presented to highlight observed patterns rather than to predict inoculation behavior. Furthermore, the correlation coefficients offer important insights by indicating the sensitivity of output parameters to input variations: a lower correlation coefficient signifies reduced sensitivity. Consequently, relationships exhibiting high correlation coefficients with uncontrollable input variation are not ideal for control strategies. The objective of the data analysis was then to assess whether thermal curve parameters could distinguish between the IH, IL, NH, and NL groups, despite differences in mass and pouring temperature.

#### 3.2.1. Values of T_max_ and Mass

**Figure 6:**
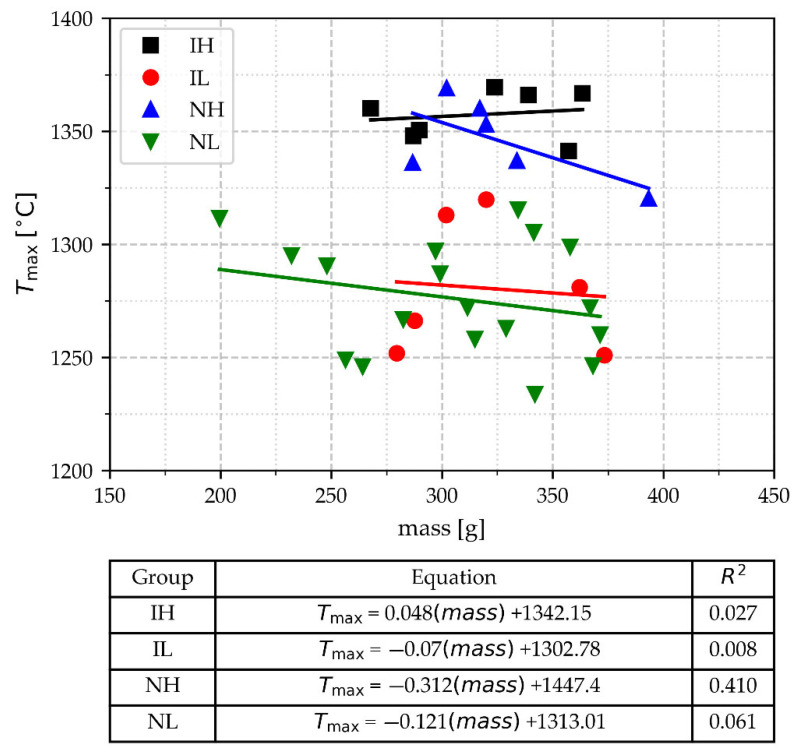
T_max_ as a function of the TA cup mass for the whole set of experiments.

The T_max_ data versus TA cups mass and the correlation lines for each group are shown in Figure 6. It is noticeable that the two groups poured at a high temperature, 1320–1369 °C, are separated from those groups poured at a low temperature, except for one T_max_ value from the high-temperature group that overlaps with the boundary of the other groups. These results support that the cups were effectively poured at two temperature ranges. Then, the data were grouped into two categories: high and low pouring range temperatures for subsequent data analysis. For simplicity, the results are presented in Figure 6, already categorized. This Figure also shows that values of T_max_ and mass are independent variables, as supported by the low correlation coefficients of the four groups. In this Figure, it is also noted that the data corresponding to light cups, with a mass under 268 g, do not show any tendency of the mass effect on T_max_.

#### 3.2.2. The Temperature of Austenite Solidification (TAS)

**Figure 7:**
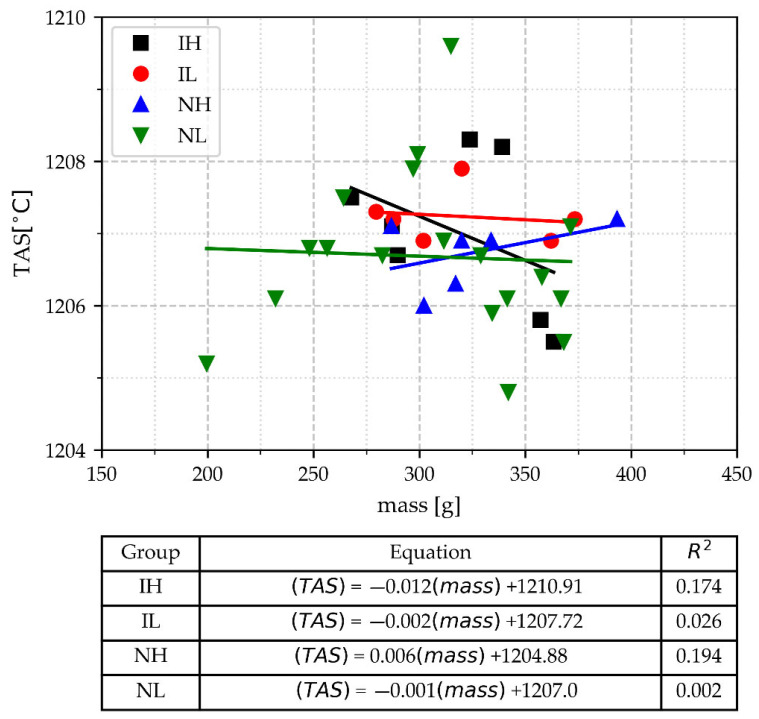
The temperature of austenite solidification, TAS, as a function of TA cup mass, pouring temperature range, and inoculation.

To determine the CE from a cooling curve, it is necessary to obtain the value of the TAS, which is possible in hypoeutectic alloys and even in light hypereutectic alloys. The values of TAS as a function of the mass, pouring temperature range, and inoculation are shown in Figure 7. In this Figure, it is shown that the TAS values vary in a range from 1210 to 1203 °C. The values of CE_AT_ calculated from these values of TAS using the equation CE_AT_ = 14.45 + 0.0089 (TAS) are in the range of 3.64–3.71% CE, while the values of CE calculated from chemical analysis are in the range of 3.67–3.72; see Table 2. Both ranges show a good agreement with each other. This finding demonstrates the robustness of TAS for determining the CE value. For practical purposes, the results show that in this case, the TAS is not significatively affected by the cup’s mass, pouring temperature, or inoculation, as also supported by the low values of the slopes and correlation coefficients of the lines obtained from the regression analysis. The insensitivity of TAS to changes in operational procedures can also be extended to the use of different types of cups, as reported by Suárez and Loper [27]. This insensitivity may be the reason why Ye et al. proposed using an even smaller cylindrical TA cup to shorten the prediction time of C and Si [34]. However, it has also been reported that TAS is sensitive to specific changes in composition [6,26]. In our experimentation, there are not enough composition variations to test this kind of sensitivity. Nevertheless, it has been reported that the determination of CE by thermal analysis is reliable due to its repeatability for a given process [26], as observed in our data.

#### 3.2.3. The Minimal Temperature of Eutectic Arrest (TE_min_)

**Figure 8:**
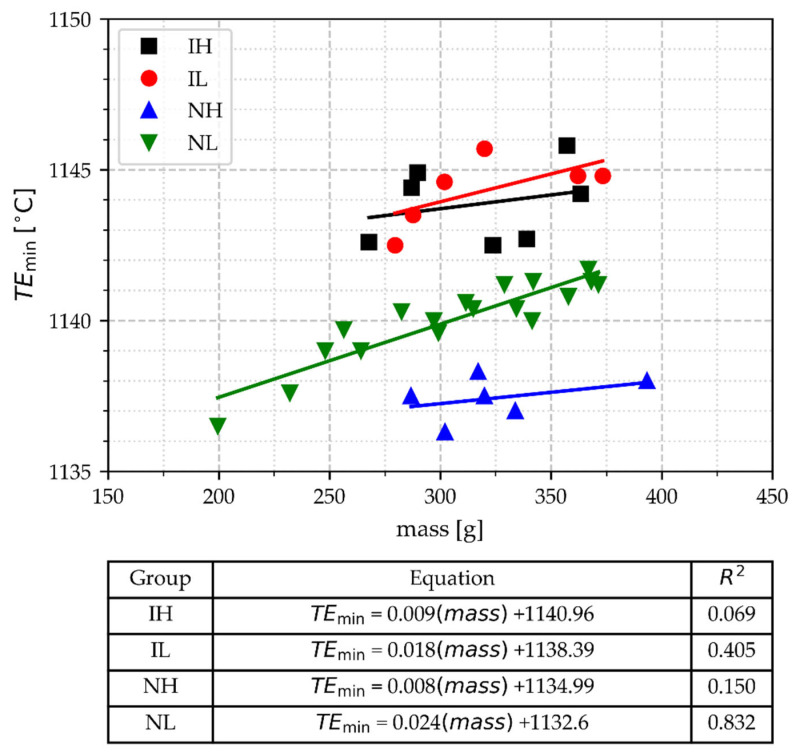
The minimal temperature of the eutectic arrest, TE_min_, as a function of TA cup mass, pouring temperature, and inoculation.

The values of the TE_min_ as a function of the TA cup mass and processing conditions are shown in Figure 8. In this Figure, it is observed that the TE_min_ values rise slightly as the TA cup mass increases; however, the slopes and correlation coefficients from the regression analysis are low, except for the data of group NL. The low correlation coefficients obtained for the groups IH, IL, and NH indicate that these groups are not significantly affected by mass values, whereas NL is. However, despite this correlation, Figure 8 shows that considering the group of cup mass NM, TE_min_ allows discrimination between inoculated (IH and IL) and non-inoculated samples (NH and NL). The values of TE_min_ for inoculated samples are in the same range, regardless of the pouring temperature. In contrast, there is a difference in the TE_min_ values of non-inoculated samples depending on their pouring temperature. High processing temperatures have been associated with low TE_min_, as is evident when comparing the two groups of non-inoculated samples (see Figure 8). At the same time, the results shown in Figure 8 indicate that inoculation eliminates this effect of the high processing temperatures.

Figure 8 shows that the values of TE_min_ for the inoculated samples are higher than 1142 °C. In the samples of the NL group, excluding the light samples (mass under 268 g), the TE_min_ values are in the range of >1139 °C and <1142 °C, whereas for the NH samples, the values are below 1139 °C. Two of the lighter cups in group NL have TE_min_ values below 1139 °C, which overlap with the TE_min_ values of NH. It is known that a high melting temperature promotes undesirable graphite morphologies in gray irons by reducing TE_min_ if not corrected with inoculation [35]. The performance of thermal analysis could be improved if this condition of overlapping, i.e., distinguishing between the NH and NL groups, could be determined from the thermal curve data. Then, avoiding light samples is suitable for achieving such improvement.

#### 3.2.4. Detection of Unsuitable Sampling

To verify whether the cooling time (CT) is a function of the cup’s mass and T_max_, it was first necessary to determine whether CT is well correlated with these variables. To estimate the relationship between cooling time, the dependent parameter, and mass and T_max_, the independent parameters, a multiple linear regression model was implemented in Python 3.10.2 using the Pandas 2.2.3 and scikit-learn 1.5.1 libraries. Experimental data were organized into a DataFrame, with mass (g) and T_max_ (°C) defined as predictor variables and CT as the response variable. The model was fitted using the ordinary least squares (OLS) method via the linear regression class, yielding the following equation:

CT = 0.4449 mass + 0.6642⋅T_max_ − 779.3205 (1)

This equation quantifies the individual influence of each variable on CT, showing a positive contribution of both mass and initial temperature to the cooling time of the metallic sample. The multiple linear regression model yielded a coefficient of determination *R*^2^ of 0.94, indicating that approximately 94% of the variance in cooling time can be explained by the mass of the sample and the initial pouring temperature.

**Figure 9:**
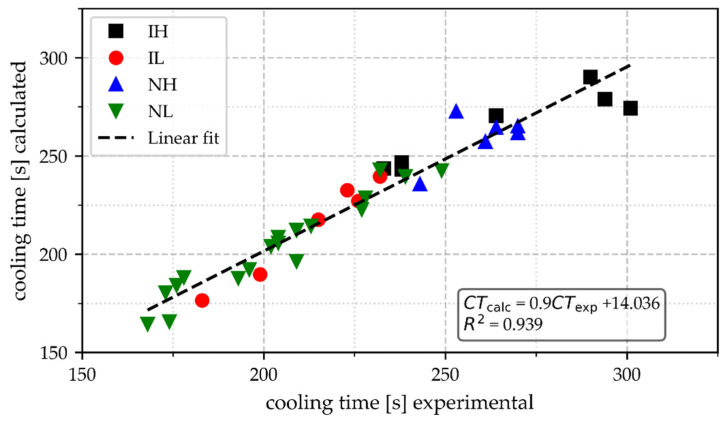
Relationship between the estimated cooling time with Equation (1) using experimental values of the cup’s mass and T_max_, and the time lapse between T_max_ and 1050 °C (CT) determined from cooling curves.

**Figure 10:**
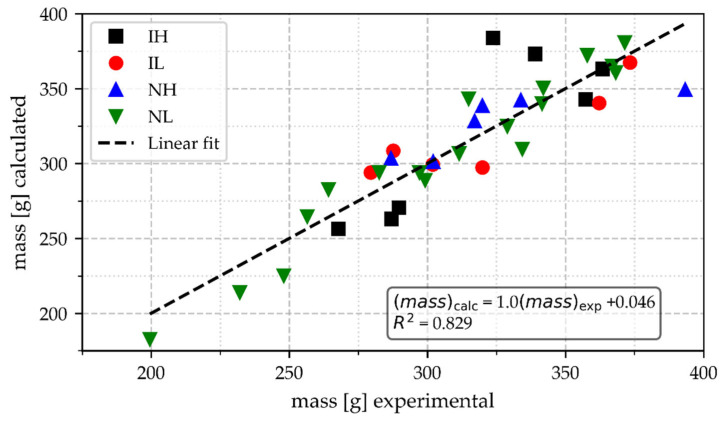
Relationship between experimental values of the mass of the cups and their cooling time.

Figure 9 shows values of calculated CT with Equation (1) as a function of experimental CT, where it is observed that there is a good correlation between such parameters, which implies that experimental CT is a good response parameter to variations in the cup’s mass and T_max_. The groups poured at high temperatures have higher CT values than the others. The difference in CT values within the groups with the same pouring temperature range must also depend on the mass of the cups, as illustrated by Figure 10. This Figure shows that light samples under 268 g and low pouring temperatures are associated with the lowest experimental values of CT, occurring under 180 s. A light sample was poured at a high pouring temperature range, and it has a CT of 215 s.

Samples with a CT under 180 s, the lighter ones in this work, poured at a low temperature, could be discarded in shop melt control, thereby improving the value of TE_min_ as a control parameter for inoculation potential. This improvement also enables TE_min_ to identify liquid metal damaged by excessively high processing temperatures, a phenomenon also reported by Riposan et al. [22], specifically the NH group in this study. The shorter cooling solidification times of round cups, compared with square cups, have affected their capability to control melt by AT, as reported by Cree et al. [15].

CT can be a good indicator of the cooling rate of the samples, as found by Zych with solidification time [15]. Zych defined the solidification time as the time elapsed from TAS to TE_ep_. CT, as described here, is independent of any parameter associated with solidification, and its determination is easier than the solidification time.

#### 3.2.5. The Maximal Temperature of the Eutectic Arrest (TE_max_)

**Figure 11:**
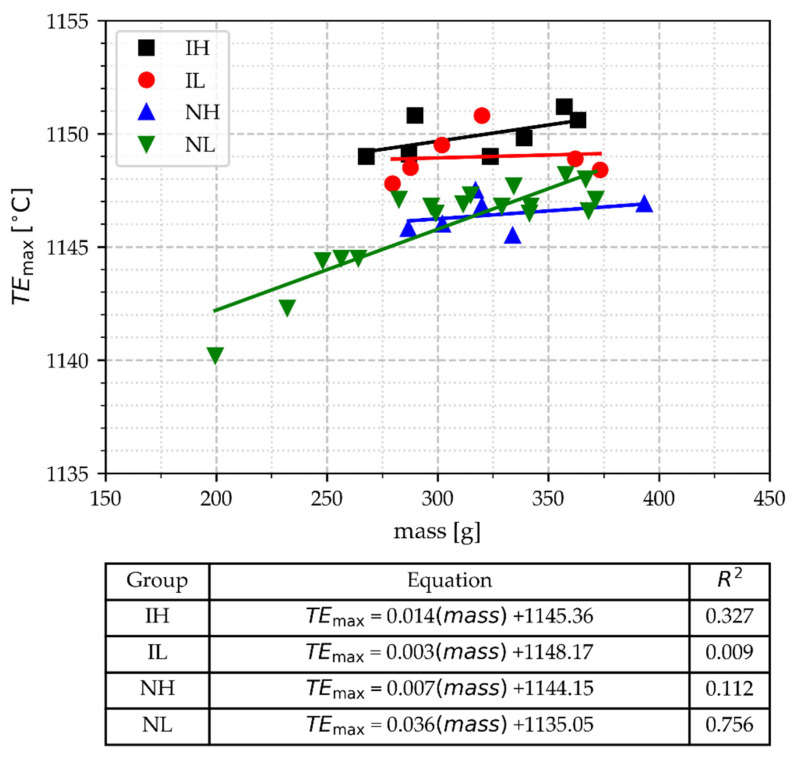
The maximal temperature of the eutectic arrest, TE_max_, as a function of TA cup mass, pouring temperature range, and inoculation.

The data of TE_max_ values grouped by inoculation and pouring temperature conditions are plotted versus TA cup mass in Figure 11. In this Figure, for the cups in the NM group, it can be observed that the inoculated groups have TE_max_ values higher than those of the non-inoculated samples. However, at around 1148 °C, there is some overlap in TE_max_ values between these groups of samples.

In the groups IH, IL, and NH, there is no substantial effect of mass on the TE_max_ values as indicated by the low regression coefficients. The NL group data show a high correlation coefficient, with slightly higher TE_max_ values than NH, except for the light cups, where the TE_max_ of NL falls below that of NH. Due to this behavior, lighter cups appear to diminish the effectiveness of TA in controlling inoculation potential.

#### 3.2.6. Recalescence (TE_max_–TE_min_)

**Figure 12:**
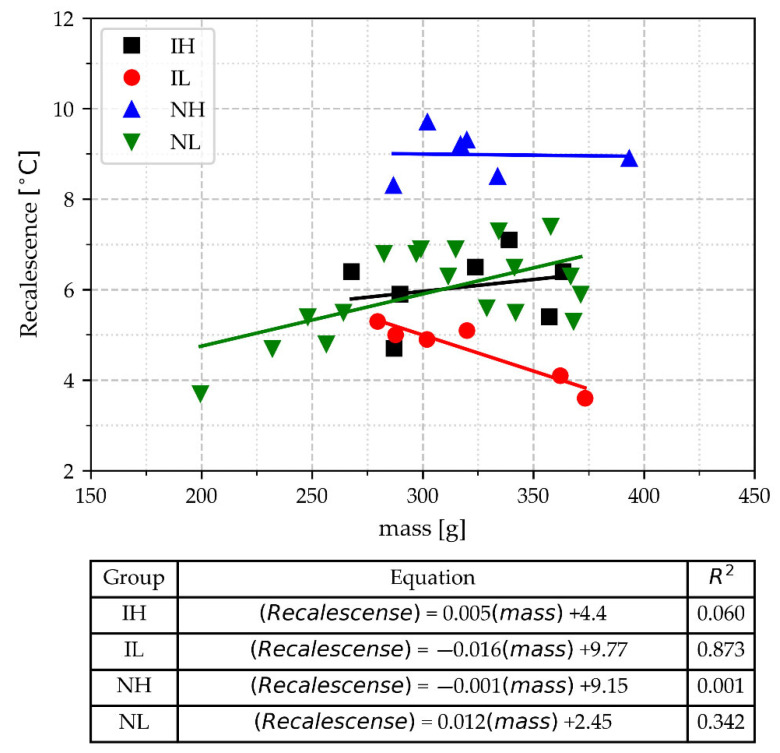
Recalescence values, TE_max_–TE_min_, as a function of TA cup mass, pouring temperature range, and inoculation.

Figure 12 shows the data for recalescence, Recal, as a function of the mass of the cups and groups according to processing variables. For this parameter, the IL group shows a higher correlation coefficient; however, this group remains distinguishable from others by Recal, except where the Recal values of NL light cups overlap. To avoid this overlap, cups weighing between 268 and 400 g are then needed to have a better-quality control parameter for inoculation potential with Recal. Considering only the NM group, it is observed in Figure 12 that the group with the worst inoculation condition, NH, is distinguished from the other groups. Recalescence values of the NH group are over eight degrees Celsius. It is also noticeable in this Figure that the group with the best inoculation condition, IL, has the lowest values of Recal, except for one overlapping point corresponding to the IH group.

#### 3.2.7. Angle on the Eutectic Deep Peak (α)

**Figure 13:**
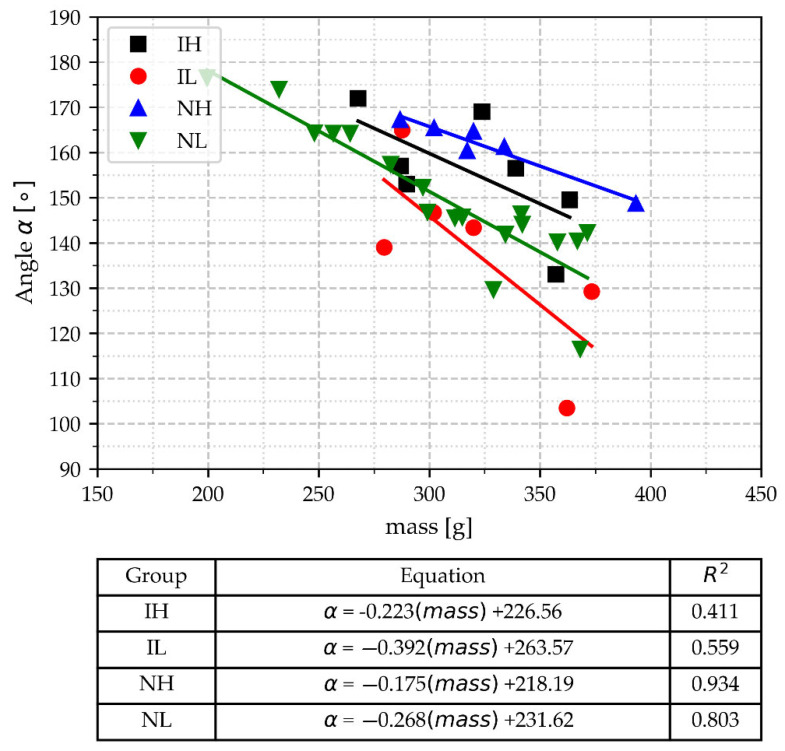
The angle a of the deep peak of the eutectic arrest as a function of TA cup mass, pouring temperature range, and inoculation.

In Figure 13, the values of the angle α are plotted vs. the cup mass. The values of the correlation coefficients for all groups are relatively high compared to the previous parameters, which means that angle α is more sensitive to the mass cup variations. Also in Figure 13, it can be observed that the points of the four groups overlap, i.e., a value of α could correspond to different groups. A notable tendency in these data is that the two groups with the high pouring temperature show higher values of the angle α compared to the other groups at the same mass values. However, as the values of the angle α do not allow discrimination between different groups and are dependent on the mass of the cups, this parameter is not suitable as a tool to control the inoculation potential in gray iron.

#### 3.2.8. The Temperature of Deep Peak (TE_ep_)

**Figure 14:**
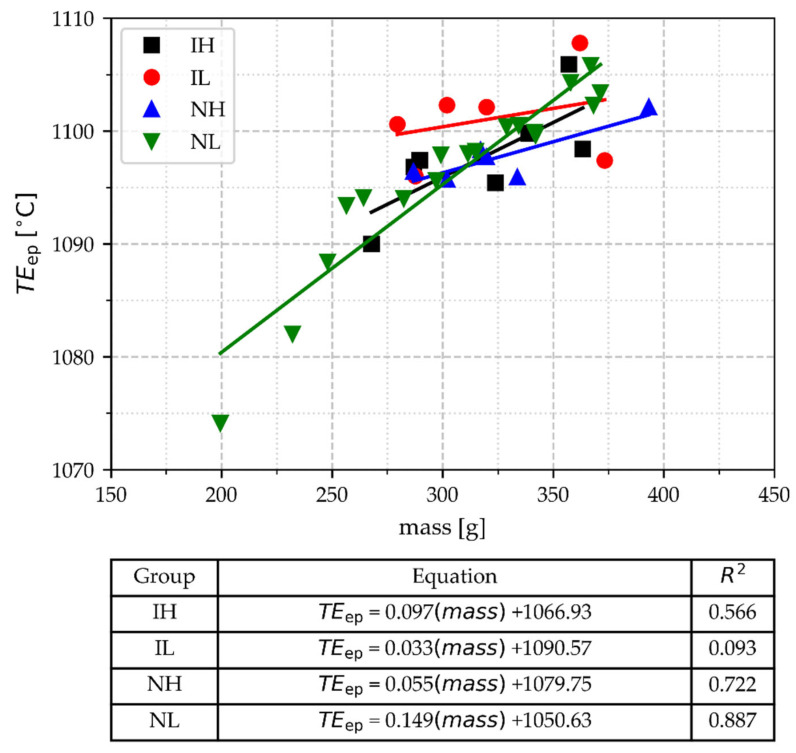
TE_ep_ of the deep peak of the eutectic arrest as a function of TA cup mass, pouring temperature range, and inoculation.

In Figure 14, the data of TE_ep_ is shown as a function of the cup’s mass. The values of TE_ep_ of all groups overlap in the range where most of the data are located, so TE_ep_ is not a suitable parameter for controlling inoculation potential in gray iron melts. However, it appears that the lighter samples with low CT can be detected with TE_ep_, whose values below 1090 °C are associated with such samples. Groups IH, NH, and NL exhibit strong correlations, indicating that TE_ep_ is generally sensitive to mass changes under the tested conditions, except group IL.

### 3.3. Microstructure

Twelve samples were chosen for cell count and visual characterization of the flake morphology. These samples were selected from the four processing conditions groups, having TE_min_ values in the range of 1136–1144 °C, and two of the samples were chosen from the light cups group.

#### 3.3.1. Graphite Flake Morphology

**Figure 15:**
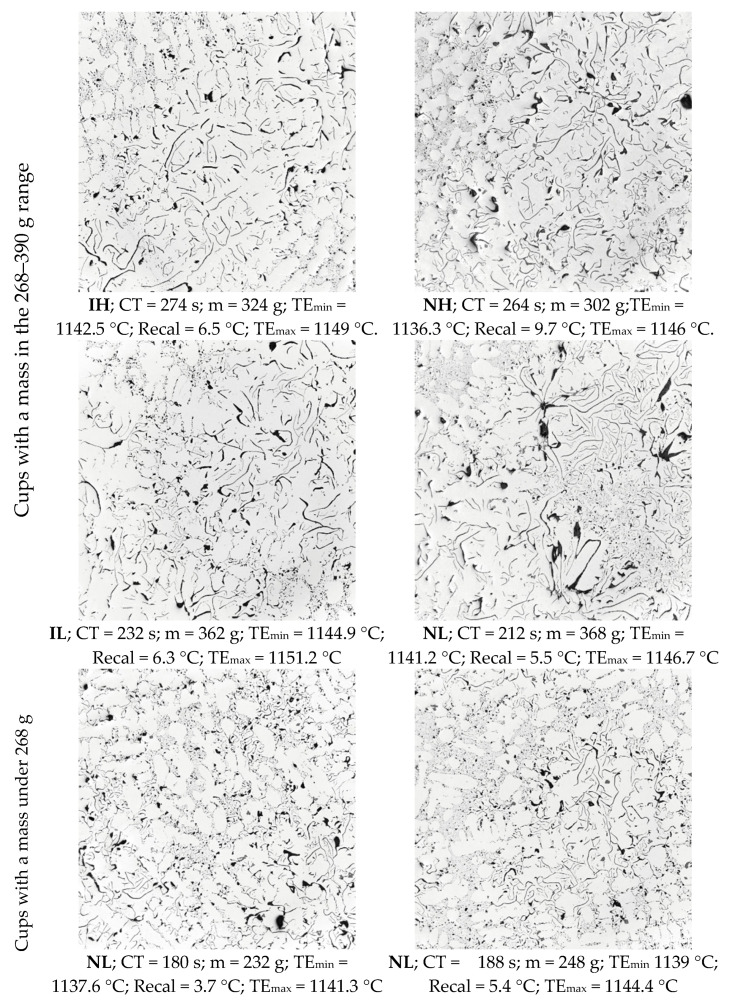
Representative micrographs of the microstructure of cups of different values of TE_min_ and processing conditions. Micrographs were acquired at 100× magnification, corresponding to a field of view measuring 1.34 mm in both height and width.

Figure 15 shows representative micrographs of TA cups with the different processing conditions. Firstly, graphite flake morphology was visually classified according to the ASTM 247 standard [36]. In this standard, graphite distribution A is related to well-shaped graphite lamellas randomly distributed, while distribution B is characterized by graphite lamellas more radially distributed around the center of a cell. Distributions D and E correspond to smaller graphite particles located between the dendrite arm spaces. Distribution D is observed as fine graphite particles, while distribution E appears as fine graphite lamellas.

The micrographs corresponding to NH and NL samples show graphite flakes with a distribution B and zones of graphite distribution D, whereas inoculated samples have more graphite flakes of distribution A. The IH sample micrograph exhibits zones of graphite distribution D, whereas the one of IL shows zones of small and thin flakes. In the samples of low mass and CT, the graphite flakes are also small and thin, and the micrographs show a higher presence of graphite distribution D.

These morphological changes can be qualitatively explained by theoretical models of eutectic growth [37,38,39], where the spacing between eutectic lamellas directly depends on the growth’s undercooling. The difference between the eutectic temperature of the melt and the temperature of the eutectic arrest determines this undercooling. In hypoeutectic gray irons, as studied here, during the cooling at some moment after the austenite formation, the liquid reaches the eutectic composition [40]. Because the composition of our samples is nearly the same, it can be supposed that this eutectic composition and, consequently, the eutectic temperature are still almost constant among the whole set of samples. This nearly constant eutectic temperature allows for a relationship between the temperature of the eutectic arrest and lamella spacing, i.e., this spacing is reduced as the temperature of the eutectic arrest decreases. Because the eutectic arrest does not have a constant temperature, the value of TE_min_ can only be chosen as a parameter characterizing such an arrest.

Theoretically, as TE_min_ increases, it is expected that the spacing between the flakes and their thickness will decrease, and the graphite distribution will transition from D, E, B, and A successively. The micrographs in Figure 15 suggest two main eutectic solidification steps. The biggest flakes of graphite are formed in the first step and can be distinguished from the small graphite particles formed in the second step. The bigger flakes in each of the micrographs of Figure 15 show that these flakes increase in spacing and thickness when TE_min_ increases.

The appearance of islands of graphite distribution E and D can occur at the second and the last stage of solidification, which can take place under temperatures below TE_min_ values at the moments when the cooling rate is also increased. At this stage, secondary nucleation can also occur [40,41,42]. The presence of islands of this type of graphite distribution tends to increase when TE_min_ decreases, as suggested by the micrographs of Figure 15.

#### 3.3.2. Cell Count

**Figure 16:**
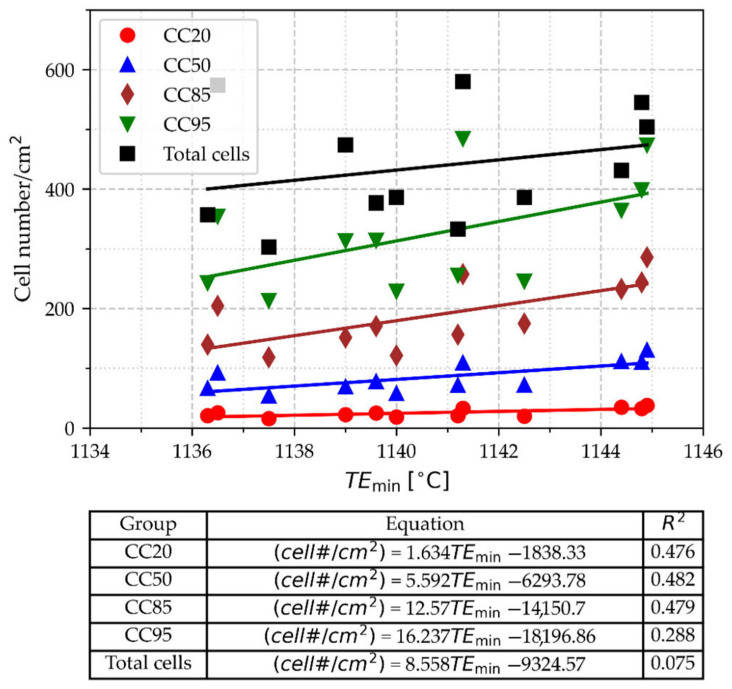
Cell numbers by square centimeter according to different count criteria: CC100; the total number of cells; and the number of cells that represent 95, 80, 50, and 20% of the total surface of cells CC95, CC80, CC50, and CC20, respectively.

The values of eutectic cell count as a function of TE_min_ are shown in Figure 16. The eutectic cell count (CC) is reported according to different criteria: (a) all cells observed on the measured surface CC100 and (b) the number of cells that represent 95, 80, 50, and 20% of the total surface of cells CC95, CC80, CC50, and CC20. To obtain these numbers, an accumulative distribution of the surface of cells from the biggest cell to the smallest one was performed. By doing this, the smaller cells are excluded from the cell count. In Figure 16, it is observed that the cell count increases as TE_min_ rises, as supported by the slopes of the regression lines.

The slopes and correlation coefficients of the linear regressions are lower than 0.5 but provide insights into the relationships between TE_min_ and cell count. The smaller correlation coefficient corresponds to the CC100-TE_min_ data, suggesting that an additional factor could increase the scatter of the data. This factor could be that in CC100, all the smaller cells are included, which may appear at the end of solidification due to secondary nucleation. Theoretically, at the time when TE_min_ was registered, these small cells had not been formed yet; therefore, they did not have any relationship with TE_min_. When the small cells are excluded from the cell count, higher correlation coefficients are obtained, which supports the idea of secondary nucleation taking place. Considering that the slope of the regression line indicates the strength between variables, the weakest relationship is between TE_min_ and CC20, which suggests that there is a loss of information if the cell count only considers the bigger cells that represent 20% of the cells’ surface

#### 3.3.3. Statistical Analysis of Cell Count

A statistical analysis of the size distribution of eutectic cells was performed for samples from all studied groups: IH, IL, NH, and NL. To conduct this analysis, it was necessary to summarize the available data from samples of each group.

The descriptive statistics are summarized in Table 3, where it is seen that the density of eutectic cells (#cells/cm^2^) is 366 and 441 for inoculated cases (IH, IL) and 275 and 446 for non-inoculated cases (NH, NL). Typically, inoculated samples are expected to have a higher cell count; however, the NL group shows a cell count higher than that of the IH group. Note, however, that the median area (Q2) is smaller for non-inoculated cases than for inoculated cases, i.e., the proportion of “small” eutectic cells is higher for the non-inoculated cases.

**Figure 17:**
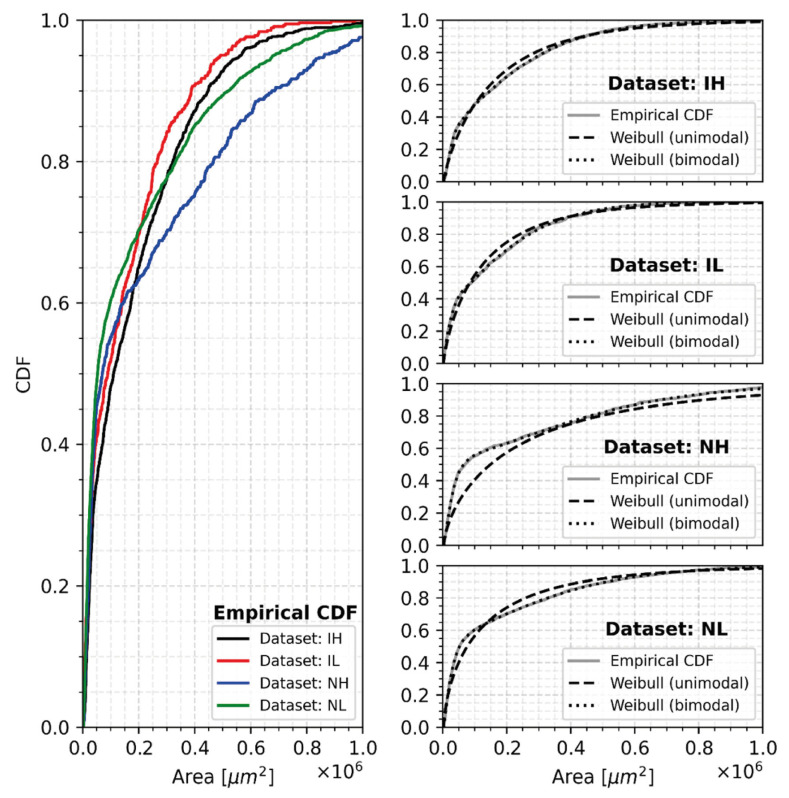
Graphical comparison of the empirical CDF and the fitted three-parameter Weibull and mixture of two three-parameter Weibull distributions.

To elucidate whether this behavior is due to a possible bimodal distribution arising from secondary nucleation, the empirical cumulative distribution functions (ECDFs) of eutectic cell sizes were calculated. Notice on the left of Figure 17 that the ECDF curves have initially steep slopes that afterward transition into a gradually flattening slope as the CDF value increases and that the endpoint of the initial steep slopes occurs at larger CDF values for non-inoculated groups than for inoculated groups, i.e., the cumulative probability of “small” size eutectic cells is larger for non-inoculated samples than for inoculated samples. The transition of decreasing rate of the slopes could hint at bimodal behavior in the eutectic cell size distributions.

To test the bimodal distribution hypothesis, the data were fitted with two statistical models (unimodal and bimodal Weibull distributions, Equations (1) and (2), respectively) using Maximum Likelihood methods [43] (pp. 403–454), and the goodness of fit was tested with the Kolmogorov–Smirnov (KS) statistic [43] (pp. 651–667), in which the null hypothesis states that the sampled data (the area measurements) come from the fitted continuous distribution. The fitted distribution parameters, along with the p-value of the KS statistics, are presented in Table 4 and Table 5 for the unimodal and bimodal models, respectively. For all cases, the p-values of unimodal models are less than 0.05 (null hypothesis is rejected), whereas bimodal models are substantially larger than 0.05 (null hypothesis is accepted).

(2) $$p\left(x,k,\sigma ,\mu \right)=\frac{k}{\sigma }{\left(\frac{x-\mu }{\sigma }\right)}^{k-1}\exp\left(-{\left(\frac{x-\mu }{\sigma }\right)}^{k}\right)$$

(3) $$p\left(x,\;\phi ,\;k_{1},\;\sigma_{1},\;\mu_{1},\;k_{2},\;\sigma_{2},\;\mu_{2}\right)=\phi \;p_{1}\left(x,\;k_{1},\;\sigma_{1},\;\mu_{1}\right)\;+\;\left(1-\phi \right)\;p_{2}\left(x,\;k_{2},\;\sigma_{2},\;\mu_{2}\right)\;p_{1}\left(x,k_{1},\sigma_{1},\mu_{1}\right)=\frac{k_{1}}{\sigma_{1}}{\left(\frac{x-\mu_{1}}{\sigma_{1}}\right)}^{k_{1}-1}\exp\left(-{\left(\frac{x-\mu_{1}}{\sigma_{1}}\right)}^{k_{1}}\right)\;p_{2}\left(x,k_{2},\sigma_{2},\mu_{2}\right)=\frac{k_{2}}{\sigma_{2}}{\left(\frac{x-\mu_{2}}{\sigma_{2}}\right)}^{k_{2}-1}\exp\left(-{\left(\frac{x-\mu_{2}}{\sigma_{2}}\right)}^{k_{2}}\right)$$

On the right of Figure 17, the cumulative distribution functions of the two fitted statistical models (unimodal and bimodal Weibull distributions) are plotted. The better suitability of the bimodal Weibull model is straight away apparent in this graphical comparison, since their CDFs overlap with the ECDFs.

The fitted weight parameters, *ϕ*, of the bimodal Weibull distributions (see Table 3) reflect this behavior as well since *ϕ* is smaller for inoculated samples (0.42 to 0.53) than for non-inoculated samples (0.55 to 0.60). Also, notice that the fitted scale parameters, *σ_2_*, of the mixture distributions (see Table 3) are larger for non-inoculated samples. These results support the idea that secondary nucleation can be more profuse in non-inoculated samples because the population of small cells is more important than that of large cells in those samples, as compared to the inoculated samples. This finding suggests that the cutting criteria for small cells should not be the same across different samples. The population of small cells can introduce scattering when searching for the relationship between cell count and TE_min_.

## 4. Summary and Conclusions

Experiments on thermal analysis (TA) were conducted with marginal variations in the chemical composition of the melt and significant changes in pouring temperature, mass of TA cups, and with or without inoculation. The high pouring temperature ranges from 1320 °C to 1369 °C, while the low range is between 1220 °C and 1320 °C. The mass of the main group of samples varied between 268 and 390 g. Five cups were poured with a mass under 268 g, non-inoculated, and had a low pouring temperature range. From the results, the insight provided by this study is to avoid mass cup under 268 g, which gives a CT under 180 s. The following conclusions were obtained:

- The capability of the temperature of austenite solidification (TAS) for estimating CE is not affected by the pouring temperature (T_max_), inoculation, or the cup’s mass, even if the cups are from the group with a lighter mass. The values of the CE estimated with the TAS (CE_TA_) are placed into a nearly identical range of values as compared with CE values obtained by chemical analysis.
- TE_min_ was able to discriminate the group of inoculated cups from non-inoculated cups. TE_min_ values for inoculated samples filled at low and at high pouring temperatures overlap. In contrast, the TE_min_ allows for distinguishing the non-inoculated samples by their pouring temperature range, except for two data points corresponding to lighter cups.
- The results showed that TE_max_ allows for the distinction between cups inoculated and those not inoculated, with some overlapping in its boundary. However, in both types of samples—those inoculated and those not inoculated—it is not possible to distinguish between those poured at high and low temperatures. Lighter samples with cooling times under 180 s are related to the lowest values of TE_max_.
- In cups with a mass range of 268–390 g, the recalescence (Recal) can distinguish the samples that are non-inoculated and poured at a high-temperature range from the others. It can even discriminate between the group of inoculated samples poured at a low-temperature range and the samples of other processing conditions, except for three non-inoculated light samples poured at a low-temperature range, which have recalescence values that overlap with this group. This overlap hinders Recal’s ability to detect poor inoculation conditions in the melt because these non-inoculated light samples are confused with those of good inoculation conditions.
- Parameters related to the deep peak at the end of the eutectic arrest angle α and TE_ep_ are very dependent on the cups’ mass, i.e., there is not any value of these parameters that allows them to distinguish between samples with different processing conditions, except that the lowest values of TE_ep_ are associated to the lightest three samples.
- The mass of the cups and T_max_ are well related to the cooling time as defined here. Light samples poured at a low-temperature range are associated with cooling time (CT) values of less than 180 s. These CTs should be avoided when TA is intended for controlling inoculation potential.
- Visual inspection of selected samples shows that inoculation is also related to the formation of graphite distribution A, and graphite distribution B tends to appear more likely in non-inoculated samples. The appearance of islands of graphite D and E also seems to increase as TE_min_ decreases.
- The distribution of cells according to their surface corresponds well to a bimodal one; the distribution of the population of the small cells is more important in the non-inoculated samples than in the inoculated ones.
- This result suggests that the cutting criteria value for cell count is not constant across the entire set of samples. However, a reasonable approach is to cut the smaller cells, which represent around 15% of the total cell surface area, to mitigate the effect of small cells on the relationship between CC and TE_min_.

## Figures and Tables

**Table 1:** Design of the experiments for thermal analysis of the solidification of gray iron, varying the cup’s mass, pouring temperature, and inoculation level.

Test	High Temperature			Test	Low Temperature		
H1	FI	NF-I	F-NI	L1	F-I	NF-I	F-NI
H2	NF-NI	F-NI	NF-I	L2	NF-NI	F-NI	NF-I
H3	F-I	NF-NI	F-NI	L3	F-I	NF-NI	F-NI
H4	F-I	NF-NI	NF-I	L4	F-I	NF-NI	NF-I

**Table 2:** Chemical composition of base metal. Carbon and silicon contents were obtained from combustion analysis with infrared absorption and the concentration of the rest of the elements by spark emission spectroscopy. * CE calculated by %C + %Si/4, and ** CE_AT_ = 14.45 + 0.0089 (TAS) [2].

	HEAT	C	Si	Mn	S	P	Cu	Pb	Zn	Ti	Al	B	Cr	CE *	CE_AT_ **
Base metal	M1	3.28	1.92	0.43	0.143	0.04	0.039	0.010	0.029	0.01	0.003	0.004	0.042	3.78	-
High temperature	H1	3.22	1.88	0.42	0.141	0.035	0.041	0.010	0.0027	0.005	0.002	0.00402	0.042	3.71	3.691
	H2	3.23	1.9	0.42	0.142	0.036	0.039	0.010	0.0028	0.005	0.002	0.00402	0.040	3.72	3.721
	H3	3.20	1.89	0.42	0.142	0.036	0.040	0.010	0.0290	0.005	0.002	0.00402	0.042	3.69	3.707
	H4	3.22	1.89	0.39	0.140	0.036	0.041	0.010	0.0028	0.004	0.002	0.00359	0.040	3.71	3.706
Low temperature	L1	3.24	1.89	0.41	0.144	0.035	0.042	0.011	0.0029	0.005	0.002	0.00356	0.040	3.73	3.716
	L2	3.25	1.89	0.4	0.144	0.035	0.040	0.011	0.0030	0.004	0.002	0.00392	0.045	3.74	3.713
	L3	3.25	1.88	0.41	0.142	0.034	0.039	0.010	0.0280	0.005	0.002	0.0037	0.040	3.74	3.709
	L4	3.24	1.92	0.42	0.142	0.036	0.040	0.012	0.0290	0.005	0.002	0.00422	0.050	3.74	3.710

**Table 3:** Number of eutectic cells per unit surface (#/cm^2^) and descriptive statistics of the eutectic cell area distribution (mm^2^): mean, standard deviation, quantiles (Q1, Q2 (median), Q3), minimum, and maximum for the four processing conditions groups.

Group	#/cm^2^	Mean mm^2^	Std. mm^2^	Min mm^2^	Q1 mm^2^	Q2 mm^2^	Q3 mm^2^	Max mm^2^
IH	366	183,341	199,888	3071	30,946	110,390	277,944	2,049,441
IL	451	151,918	166,856	3014	22,372	89,510	234,114	972,272
NH	275	234,204	303,243	3424	24,138	70,314	393,676	1,940,605
NL	446	173,347	231,299	3003	19,658	53,305	260,849	1,474,339

**Table 4:** Fitted unimodal Weibull distribution (Equation (2)) parameters and results of the Kolmogorov–Smirnov goodness-of-fit test; *p*-values < 0.05 hint that the underlying eutectic cell area distributions do not follow unimodal Weibull distributions.

	Distribution Parameters *			K-S Test
Group	*k*	*σ*	*μ*	*p-Value*
IH	0.8385	162,657	3071	1.7 × 10^−5^
IL	0.7927	130,849	3015	0.01
NH	0.6976	247,702	3423	7.3 × 10^−21^
NL	0.6786	127,423	3003	8.9 × 10^−23^

* *k*: shape parameter, *σ*: scale parameter, *μ*: location parameter.

**Table 5:** Fitted bimodal Weibull distribution (Equation (3)) parameters and results of the Kolmogorov–Smirnov goodness-of-fit test; *p*-values > 0.05 hint that the underlying eutectic cell area distributions follow bimodal Weibull distributions.

	Distribution Parameters **							K-S Test
Group	*ϕ*	*k_1_*	*σ_1_*	*μ_1_*	*k_2_*	*σ_2_*	*μ_2_*	*p-Value*
IH	0.4208	1.2376	28,161	4738	1.229	244,779	61,184	0.9988
IL	0.5338	0.9538	31,471	2839	1.28	213,581	84,665	0.9998
NH	0.5576	1.1842	31,354	3644	1.3948	465,811	65,456	0.9896
NL	0.6084	1.0494	29,175	4161	1.2955	344,638	75,538	0.5489

** *ϕ*: weight parameter, *k*: shape parameter, *σ*: scale parameter, *μ*: location parameter.

## Data Availability

All original data of this research were included in this article. Further inquiries can be directed to the corresponding author.

## Abbreviations

The following abbreviations are used in this manuscript:

TA	Thermal analysis
CE	Carbon equivalent
FI	Full cup, inoculated
NF NI	Non-full cup, non-inoculated
NM	Normal mass range
NH	Non-inoculated high temperature
IL	Inoculated low temperature
DA_max_	First derivative maximum point
TAS	Austenite solidification temperature
TL	Liquidus temperature
DE_min_	First zero crossing of the first derivative at the eutectic arrest
DE_max_	Second zero crossing of the first derivative at the eutectic arrest
DE_ep_	Minimum point of the final negative peak in the first derivative at the eutectic arrest
TE_min_	Temperature corresponding to the DE_min_
TE_max_	Temperature corresponding to the DE_max_
TE_ep_	Temperature corresponding to the DE_ep_
T_max_	Peak temperature measured by the thermocouple after pouring
CT	Cooling time (time interval between T_max_ and 1050 °C)
CE_AT_	Carbon equivalent determined by thermal analysis
OLS	Ordinary least squares
CC	Eutectic cell count
ECDF	Empirical cumulative distribution functions
*ϕ*	Weight parameter, bimodal distribution
*k*	Shape parameter, Weibull distribution
*σ*	Scale parameter, Weibull distribution
*μ*	Location parameter, Weibull distribution
